# Alkaloid leonurine exerts anti‐inflammatory effects via modulating MST1 expression in trophoblast cells

**DOI:** 10.1002/iid3.493

**Published:** 2021-07-28

**Authors:** Fang Zong, Yingzi Zhao

**Affiliations:** ^1^ Department 3 of Obstetrics Cangzhou Central Hospital Cangzhou China

**Keywords:** leonurine, MST1, NF‐κB signal pathway, trophoblast cells

## Abstract

**Background:**

Pre‐eclampsia (PE) is mainly attributed to the inflammation of trophoblast cells in pregnant women, which results in damage to the maternal organs and growth retardation of the fetus. Alkaloid leonurine (LNR) is a plant compound and has anti‐inflammatory effects. Here we aimed to investigate the effects of LNR on human and mouse trophoblast cells and the underlying mechanisms.

**Methods:**

The levels of the inflammatory factors in trophoblast cells under lipopolysaccharides (LPS) stimulation were analyzed with ELISA. Western blot was employed to examine the protein expression. Trophoblast cells in Mammalian ste20‐like kinase 1 (MST1^−/−^) or wild type (WT) mice were isolated to examine the expression of signal molecules in the nuclear factor‐κB (NF‐κB) pathway. Concentration‐dependent activity of NF‐κB was examined. The regulation of LNR and MST1 in MST1^−/−^ trophoblast cells was studied as well.

**Results:**

Our data showed that LNR exhibited anti‐inflammatory effects and suppressed the NF‐κB signaling by inhibiting LPS‐induced inflammation in trophoblast cells. LNR upregulated the expression of MST1, and the anti‐inflammatory role of LNR was greatly relieved in MST1‐knockout trophoblast cells, although it displayed weak roles in NF‐κB signaling.

**Conclusion:**

LNR exhibits anti‐inflammatory effects on human and mouse trophoblast cells by upregulating MST1 in the NF‐κB signal pathway.

## Graphical abstract

Leonurine (LNR) exhibited anti‐inflammatory effects and suppressed the NF‐κB signaling by inhibiting LPS‐induced inflammation in trophoblast cells. LNR played its anti‐inflammation effects on human and mouse trophoblast cells by upregulating MST1 in the NF‐κB signal pathway. LNR, leonurine; LPS, lipopolysaccharides; NF‐κB, nuclear factor kB.

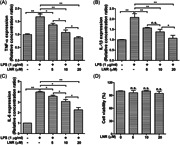

## 1 INTRODUCTION

Trophoblasts provide nutrients to the embryo and develop into a large part of the placenta. They play an important role in embryo implantation and interaction with the decidualized maternal uterus.^1^, ^2^ The placenta then facilitates the exchange of nutrients, wastes, and gases between the maternal and fetal systems. As the placenta is a highly invasive organ, the reproduction, migration and invasion of the trophoblasts during the development of the placenta must be precisely controlled to ensure sufficient and adequate nutrient supply.^3^, ^4^, ^5^ However, abnormal conditions, such as oxidative stress, proinflammatory cytokines and auto‐antibodies, induce chronic inflammation of the placenta, which influences placental function and often results in pre‐eclampsia (PE).^6^, ^7^ The reduced permeability of human trophoblasts can interfere with arterial weight. These conditions lead to hypoxia in the placenta, resulting in increased reactive oxygen species or lack of 5'‐Adenylate triphosphate. Placental oxidative stress in turn leads to excessive release of inflammatory factors, leading to pregnancy disorders. As a result, research on the pathogenesis of PE mainly focuses on trophoblast cells.^8^ Therefore, studying the anti‐inflammatory mechanism in trophoblast cells is very important for the prevention of maternal and fetal diseases.

Mammalian ste20‐like kinase 1 (MST1) is a component of the tumor necrosis factor α (TNF‐α) receptor 1 signaling complex and attenuates TNFα‐induced NF‐κB signaling, which in turn plays a central role in inflammatory and immune responses.^9^ MST1 is generally expressed in apoptosis‐promoting kinase, and recently, it is found that inhibition or loss of function of MST1 promotes breast cancer proliferation,^10^ as well as protects mouse embryos.^11^ Thus, MST1 possesses important roles in anti‐inflammation and cancer suppression.

Studies have shown that many plant extracts exhibit anti‐inflammatory and antioxidative properties.^12^ Leonurine (LNR) is one of the active plant compounds from Herba leonuri. LNR blocks the phosphorylation of NF‐κB and reduces the inflammatory response in the kidney.^13^ Other researchers have demonstrated that LNR exerts cardio‐protective effects and slows down lipopolysaccharides (LPS)‐induced inflammation, possibly by blocking the NF‐кB pathway.^14^, ^15^ However, the effects of LNR on trophoblast cells and its target genes have not been fully investigated. In this study, we hypothesized that LNR could exert anti‐inflammatory effects in LPS‐induced inflammation in trophoblast cell model.

## 2 MATERIALS AND METHODS

### 2.1 Cell culture

The human placental trophoblast cells HTR8/Svneo was purchased from American Type Culture Collection (ATCC CRL‐3271). Cells were cultured in ATCC‐formulated RPMI‐1640 Medium, supplemented with 10% fetal bovine serum (FBS; Sigma‐Aldrich) and 1% penicillin/streptomycin (Gibco). When the cells reached 85% confluency, they were trypsinized with 1× trypsin‐EDTA (Sigma) and passaged with 1:3 dilution in a new 75 cm^2^ flask. LNR LPS were purchased from Sigma‐Aldrich. For LPS‐induced inflammatory model, HTR8/Svneo cells were cultured in a six‐well plate, when cells reached 70%–80% confluency, different concentrations of LNR (0, 5, 10 and 20 μM) were added in each relative well and incubated at 37°C for 6 h. The concentrations of LNR were chosen based on the previous publication.^14^ A volume of 1 mg/ml of LPS was then added to relative wells according to experiment design. Cells were incubated for another 6 h. After a total of 12‐h incubation, cells were considered ready to be harvested for further experiments.^2^

### 2.2 Animals

The wild type (WT) and MST1^+/−^ mice with C57BL/6 background were purchased from GemPharmatech. The animals were kept under temperature‐ and humidity‐controlled condition (the temperature at 22°C–24°C and humidity at 60% ± 5%). The MST1^+/−^ mice were bred in a specific pathogen‐free facility. Animal studies were approved by the Ethics Committee of Cangzhou Central Hospital.

### 2.3 Collection of trophoblast cells from WT or MST1 knockout (MST1^−/−^) mice

For trophoblast cell collection, late‐stage pregnant mice were euthanized. The uteri were removed and the placenta were carefully separated and were placed into a 10 ml sterile tube with plain Dulbecco's modified Eagle medium media, with each tube containing a single placenta. The tissue was cut and ground with a grinder, then the cell suspension was pipetted and centrifuged, and the middle layer containing trophoblast cells was pipetted and washed with phosphate‐buffered solution. Trophoblast cells were cultured in NCTC‐135 medium. Polymerase chain reaction analysis was applied for genotyping. Only the MST1^−/−^ genotype and WT were kept for further use.^11^, ^16^, ^17^

### 2.4 Western blot

Antibodies for p‐p65, NF‐κB p65, p‐IκBα, IκBα, p‐IKKα/β, and glyceraldehyde 3‐phosphate dehydrogenase were purchased from Cell Signaling Technology. MST1 antibody was purchased from Abcam. To perform Western blot, cells were harvested and lysed in 1× lysis buffer (Thermo) with 1× Protease Inhibitor Cocktail (Thermo). Proteins were measured by Micro BCA Protein Assay Kit (Thermo). Ten micrograms of total protein from each sample were resolved on 10% sodium dodecyl sulfate Bis‐Tris polyacrylamide gel and transferred onto 0.2 μm polyvinylidene fluoride membranes (Bio‐Rad). The membranes were incubated in 5% nonfat milk on a flat shaker for 1.5 h, followed by probing with primary antibody for 2 h at room temperature or overnight at 4°C. The membranes were incubated with corresponding secondary antibody for 1 h. Images were generated using an ECL system (Super Signal west Dura Kit, Thermo).^14^

### 2.5 Enzyme‐linked immunosorbent assay (ELISA)

Serum levels of TNF‐α, interleukin‐1β (IL‐1β) and IL‐6 were measured by ELISA kit from Abcam.^18^ Briefly, a 96‐well microplate for detecting each factor was prepared. A specific monoclonal antibody was precoated in each well of the plate. A volume pf 50 µl of standards, control, and samples were pipetted into the wells in duplicate. After 1 h of incubation at room temperature, washing buffer was added to wash away any unbound substances three times. Next, 100 µl of working substrate solution with equal volume of color reagents A and B was added to the wells, incubated for 20 min at room temperature with cover to avoid light, followed by adding 50 µl of Stop Solution to each well. The color development was stopped and the intensity of the color was measured by a plate reader at 410 nm, and the serum value was calculated from specific calibration curves prepared with known standard solutions.

### 2.6 Luciferase activity assay of NF‐κB p65

We cloned the 3'‐untranslated region of DDX5 into the plasmid psiCHECK2 (Promega) carrying a fluorescent reporter gene, and transfected with a plasmid containing NF‐κB p65 sequence to MST1^−/−^ trophoblast cells. After 48 h of transfection, cells were collected and the luciferase activity was measured using the manufacturer's instructions.^15^

## 3 RESULTS

### 3.1 Leonurine showed anti‐inflammatory effects in LPS‐induced inflammatory trophoblast cells

**Figure 1:**
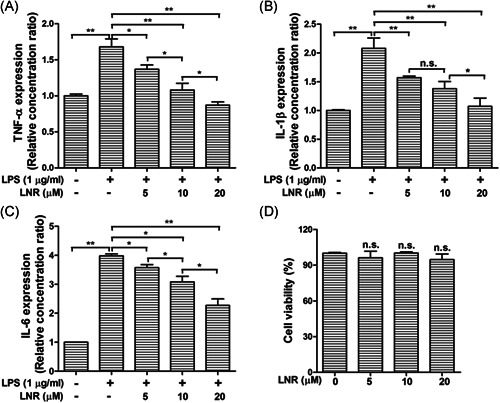
LNR alleviates LPS‐induced inflammatory cytokine production in trophoblast cells. (A–C) HTR8/SVneo cells were pretreated with LNR (0–20 μM) for 6 h, followed by stimulation with or without 1 μg/ml LPS for 6 h. The levels of TNF‐α (A), IL‐1β (B), and IL‐6 (C) in supernatant were measured by ELISA. (D) HTR8/SVneo cells were treated with 0–20 μM LNR for 12 h, and then cell viability was measured by CCK‐8 assay. IL, interleukin; LNR, leonurine; LPS, lipopolysaccharides; n.s., not significant; TNF‐α, tumor necrosis factor α;. *p < .05; **p < .01

LPS is known to induce inflammatory response in trophoblast cells. Thus, we first examined the levels of inflammatory factors TNF‐α, IL‐1β, and IL‐6 under the treatment of LNR in LPS‐induced inflammatory trophoblast cells. When the HTR8/SVneo cells were treated with different concentrations (0–20 µM) of LNR for 6 h, cells were stimulated with or without 1 µg/ml of LPS for another 6 h. With no LNR treatment, it was observed that the levels of the inflammatory factor TNF‐α was extremely high when stimulated with LPS. However, TNF‐α levels decreased gradually with increasing concentrations of LNR, and significant differences were observed (Figure 1A). Similar expression patterns were also observed for IL‐1β and IL‐6 (Figure 1B,C). The results showed that LNR possessed anti‐inflammatory effects on LPS‐induced inflammation in trophoblast cells. In addition, CCK‐8 assay showed that LNR did not affect the cell viability of HTR8/SVneo cells (Figure 1D), indicating the good biocompatibility at the observed concentrations.

### 3.2 Leonurine suppressed NF‐κB signaling by inhibiting IKK activation in LPS‐induced inflammatory trophoblast cells

**Figure 2:**
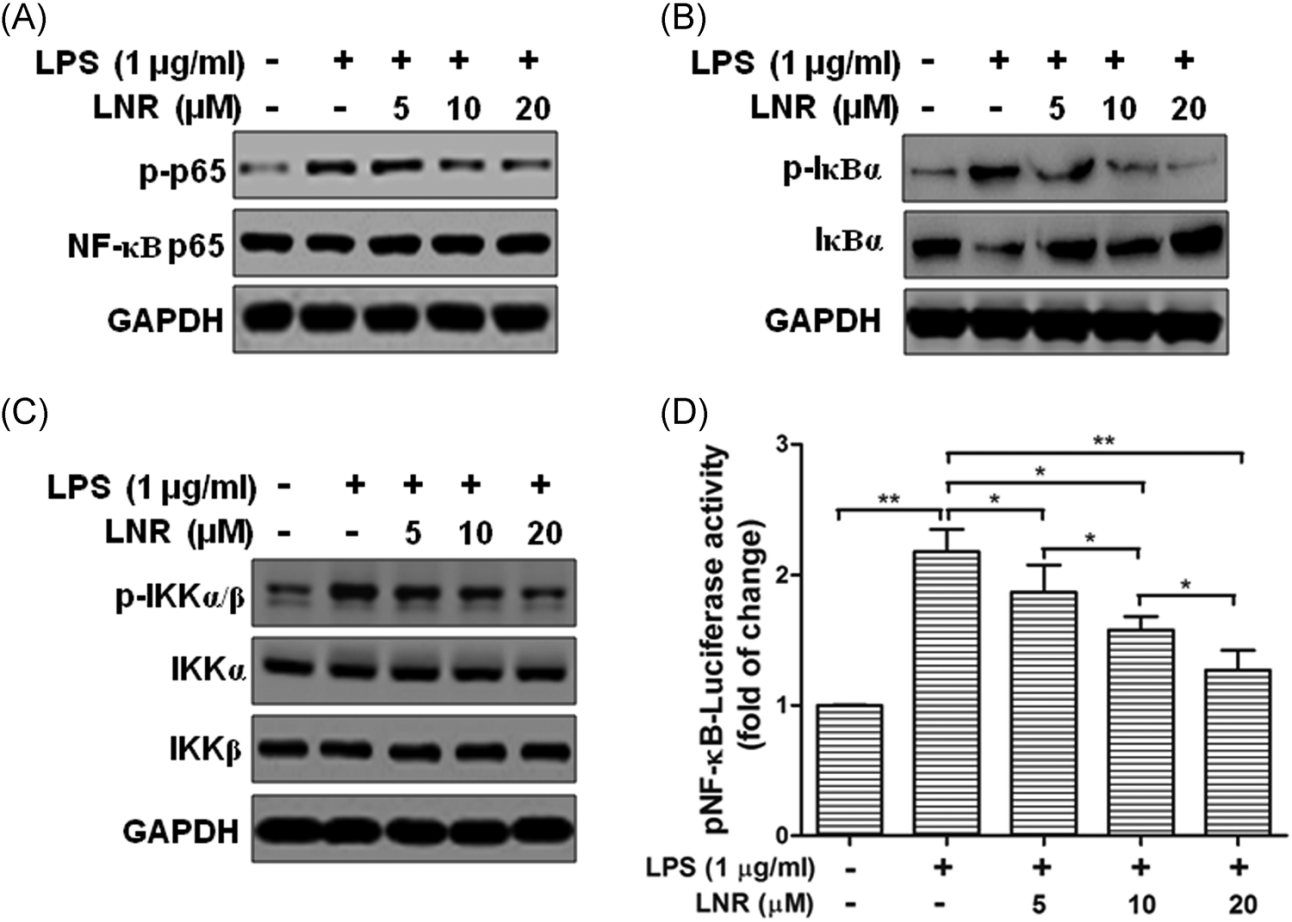
LNR inhibits NF‐κB signaling by suppressing IKK activation in LPS‐induced inflammatory trophoblast cells. (A–C) HTR8/SVneo cells were pretreated with LNR (0–20 μM) for 6 h, followed by stimulation with or without 1 μg/ml LPS for 6 h. The expression of p‐p65 and NF‐κB p65 (A), p‐IκBα and IκBα (B), and p‐IKKα/β, IKKα, and IKKβ (C) was measured by Western blot. GAPDH was used as a loading control. (D) Activity of NF‐κB after LNR treatment in HTR‐8/SVneo cells was measured by luciferase reporter assay. GAPDH, glyceraldehyde 3‐phosphate dehydrogenase; LNR, leonurine; LPS, lipopolysaccharides; NF‐κB, nuclear factor κB; TNF‐α, tumor necrosis factor α. *p < .05, **p < .01

To further examine the inflammatory regulation pathways in the LNR‐treated trophoblast cells, we checked the expression of inflammatory regulating factors in the NF‐κB signal pathway under LPS‐induced inflammatory model. The expression levels of phosphorylated NF‐κB p65, IκBα, and IKKα/β, together with their nonphosphorylated proteins, were examined using Western blot (Figure 2). The trophoblast HTR8/SVneo cells were pretreated with LNR (0–20 μM) for 6 h and then stimulated with or without 1 μg/ml LPS for another 6 h. The expression of p‐p65 exhibited low level on the condition of LPS (–)/LNR (–) (Figure 2A, first lane), then reached the highest level without LNR treatment (Figure 2A, Lane 2). The expression of p‐p65 was decreased with increasing concentrations of LNR (Figure 2A, Lanes 3–5). The phosphorylation status of IκBα and IKKα/β showed similar patterns as p‐p65 (Figure 2B,C). For the nonphosphorylated proteins, IκBα visibly exhibited expression trend opposite to its phosphorylated counterpart. However, p65, IKKα, and IKKβ did not show significant changes (Figure 2B,C). The luciferase activity assay was then performed on NF‐κB p65 protein. Results showed that the activity of NF‐κB exhibited a concentration‐dependent reduction (Figure 2D). These results suggested that the LNR suppressed the NF‐κB signaling by inhibiting IKK activation.

### 3.3 Leonurine upregulated MST1 expression in LPS‐induced inflammatory trophoblast cells

**Figure 3:**
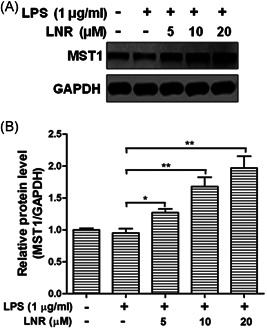
LNR upregulates the expression of MST1 in LPS‐induced inflammatory trophoblast cells. (A) HTR8/SVneo cells were pretreated with LNR (0–20 μM) for 6 h, followed by stimulation with or without 1 μg/ml LPS for 6 h. The expression of MST1 was measured by Western blot. GAPDH was used as a loading control. (B) Optical density ratio analysis. GAPDH, glyceraldehyde 3‐phosphate dehydrogenase; LNR, leonurine; LPS, lipopolysaccharides; MST1, mammalian ste20‐like kinase 1. *p < .05, **p < .01

To investigate the potential factors under LNR regulation in the NF‐κB pathway, we examined MST1 in the upstream of this pathway. MST1 is a negative regulator of NF‐κB. Under LSP‐induced inflammation, we found that the expression of MST1 increased markedly with elevated LNR (Figures 3A,B). These data indicated that LNR upregulated the expression of MST1 in LPS‐induced inflammatory HTR8/SVneo cells.

### 3.4 The anti‐inflammatory effect played by LNR was greatly compromised in MST1‐knockout mouse trophoblast cells

**Figure 4:**
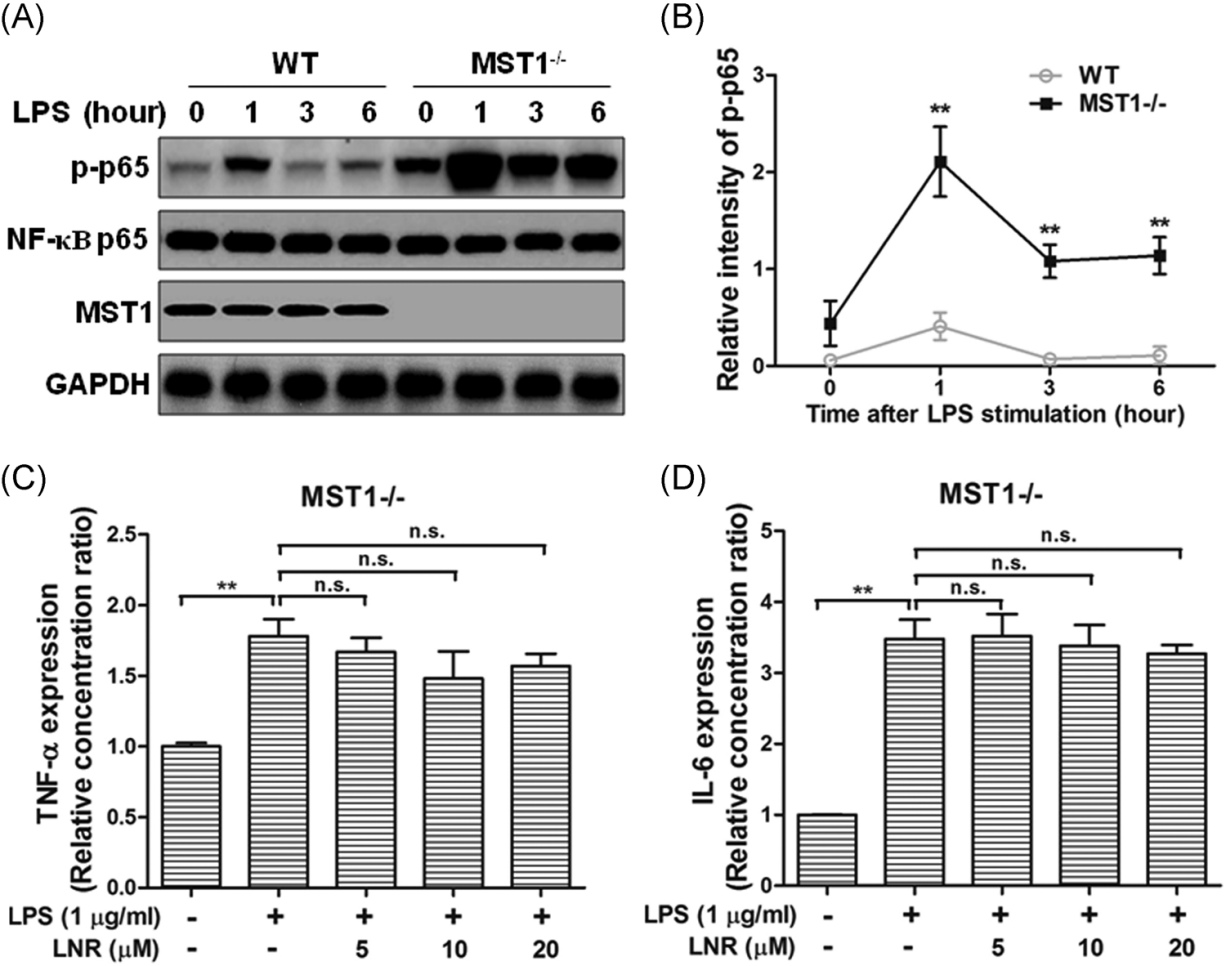
LNR plays a weak anti‐inflammatory role in MST1‐knockout trophoblast cells. (A) WT or MST1‐knockout mouse primary trophoblast cells (MST1^−/−^) exposed to 1 μg/ml LPS were immunoblotted with antibodies to p‐p65, NF‐κB p65, or MST1. GAPDH was used as a loading control. (B) Optical density ratio analysis of (A). (C,D) MST1^−/−^ cells were pretreated with LNR (0–20 μM) for 6 h, followed by stimulation with or without 1 μg/ml LPS for 6 h. The levels of TNF‐α (C) and IL‐6 (D) in supernatant were measured by ELISA. GAPDH, glyceraldehyde 3‐phosphate dehydrogenase; LNR, leonurine; LPS, lipopolysaccharides; MST1, mammalian ste20‐like kinase 1; n.s., not significant; WT, wild‐type. **p < .01,

Next, we investigated the anti‐inflammatory effects of MST1 in mouse trophoblast cells. We collected trophoblast cells from the placentas of pregnant MST1 knockout (MST1^−/−^) or WT mice. Under LPS‐induced inflammation, the protein levels of p‐p65, NF‐κB p65, and MST1 were examined at 0, 1, 3, and 6 h in HTR8/SVneo cells (Figure 4). While the trophoblast cells were treated with LNR, the expression of p‐p65 was inhibited concentration‐dependently with elevated LNR in the WT cells. Meanwhile, the inhibition of p‐p65 was greatly alleviated by MST1^−/−^ (Figure 4A,B). The inflammatory factors TNF‐α and IL‐6 levels were also examined by ELISA in MST1^−/−^ cells, and no significant differences were observed (Figure 4C,D). These results indicated that LNR played its anti‐inflammatory function by upregulating MST1 in the NF‐κB signaling pathway.

### 3.5 Leonurine displayed weak effects in NF‐κB signaling in MST1^−/−^ trophoblast cells

**Figure 5:**
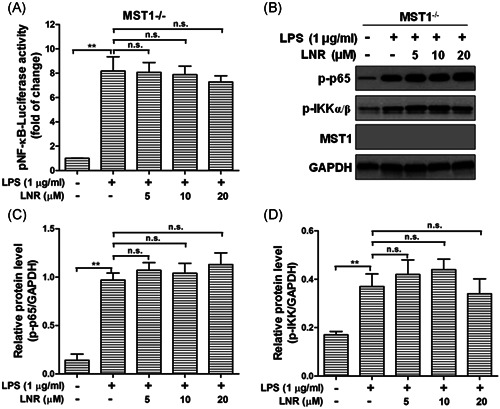
LNR displays weak roles on NF‐κB signaling in MST1‐knockout trophoblast cells. (A) Activity of NF‐κB after LNR treatment of MST1^−/−^ cells was measured by luciferase reporter assay. (B) MST1^−/−^ cells were pretreated with LNR (0–20 μM) for 6 h, followed by stimulation with or without 1 μg/ml LPS for 6 h. The expression of p‐p65, p‐IKKα/β, and MST1 was measured by Western blot. GAPDH was used as a loading control. (C,D) Optical density ratio analysis of (B). GAPDH, glyceraldehyde 3‐phosphate dehydrogenase; LPS, lipopolysaccharides; MST1, mammalian ste20‐like kinase 1; n.s., not significant. **p < .01

Finally, we examined the concentration‐dependent activity of NF‐κB after LNR treatment in MST1^−/−^ cells, as well as the protein levels of p‐p65, p‐IKKα/β (Figure 5). Interestingly, in MST1^−/−^ trophoblast cells, the activity of NF‐κB remained at high levels with increasing concentrations of LNR, no significant differences were observed (Figure 5A). The other proteins in the NF‐κB pathway, p‐p65, and p‐IKKα/β were examined by Western blot, which remained at high levels under LPS‐induced inflammation and unaffected by LNR‐treatment. No significant differences were observed (Figure 5B–D). These results indicated that LNR regulated the NF‐κB signal pathway via MST1 in mouse trophoblast cells.

## 4 DISCUSSION

LNR is a type of plant phenolic alkaloids extracted from Herba leonuri. Research has demonstrated that it plays an anti‐inflammatory role in nonalcoholic steatohepatitis,^19^ osteoarthritis,^20^ intestinal inflammation,^21^ endometritis^22^ and other inflammatory diseases. LNR displays regulatory effect in LPS‐induced inflammation models.^14^, ^23^, ^24^, ^25^ Studies showed that LNR played its anti‐inflammation role via several signal pathways, with LPS‐induced inflammation in the NF‐κB pathway being one of the commonly involved regulations.^14^, ^18^, ^23^, ^25^ However, the anti‐inflammatory function of LNR in trophoblast cells remained poorly understood.

In our study, we investigated the effects of LNR in trophoblast HTR8/SVneo cells. Our results have shown that LNR exhibited anti‐inflammatory capacity in LPS‐induced inflammatory model and suppressed the inflammatory factors TNF‐α, IL‐1β, and IL‐6 (Figure 1). Moreover, the anti‐inflammatory effect of LNR was achieved by inhibiting IKK activation in the NF‐κB signal cascade (Figure 2). Next, we searched for the potential target of LNR in the NF‐κB pathway and identified MST1, a negative regulator of NF‐κB.^9^, ^26^, ^27^ Under LPS‐induced inflammation, LNR upregulated the expression of MST1, confirming that MST1 was a target of LNR in the NF‐κB pathway (Figure 3). As the regulatory mechanism of MST1 in trophoblast cells was not elucidated, we further collected the trophoblast cells from MST1^−/−^ and WT mice, respectively. Interestingly, the MST1^−/−^ cells were more sensitive to LPS‐induced inflammation than WT cells. Moreover, LNR lost its capacity of inflammatory inhibition upon MST1 knockout, clearly indicating that LNR played its anti‐inflammatory role by regulating MST1 (Figure 4). Finally, we examined the role of LNR in MST1^−/−^ mouse trophoblast cells. As expected, LNR showed weak effects on MST1 knockout trophoblast cells, exerting little anti‐inflammatory function (Figure 5).

MST1 is a member of the Hippo pathway and plays an important role in cell growth, apoptosis and tissue regeneration, as well as a tumor suppressor.^28^, ^29^, ^30^ To date, few study has investigated LNR regulation on MST1. Although our data provide an important view supporting LNR for anti‐inflammation treatment in clinical research, some issues still remain. First, more cell lines or primary cultures of trophoblast cells could be introduced to verify the current findings. Second, it would be important to examine the effects of LNR in the disease model of animals. In addition, the detailed mechanisms of LNR regulation on MST1 are not clear: (i) Is MST1 a direct target or indirect target of lenurine, and how do they interact? (ii) Are there any other factors involved in the regulation? These questions are interesting and crucial topics of our further research, which will provide important clinical evidence concerning trophoblast cell inflammation‐induced PE.

## 5 CONCLUSION

Taken together, we conclude that LNR exerts anti‐inflammatory effects on both human and mouse trophoblast cells by upregulating MST1 in the NF‐κB pathway. Our study provides crucial significance in research and clinic for trophoblast cell inflammation‐induced PE.

## ETHICS APPROVAL

Animal studies were approved by the Ethics Committee of Cangzhou Central Hospital.
